# Microstructural Characterization of Fibric Peat Stabilized with Portland Cement and Silica Fume

**DOI:** 10.3390/ma16010018

**Published:** 2022-12-20

**Authors:** Afnan Ahmad, Muslich Hartadi Sutanto, Niraku Rosmawati Ahmad, Mazizah Ezdiani Mohamad, Mastura Bujang

**Affiliations:** 1 Department of Civil & Environmental Engineering, Universiti Teknologi PETRONAS, Seri Iskandar 32610, Perak, Malaysia; 2 Centre of Research for Innovation and Sustainable Development (CRISD), School of Engineering and Technology, University of Technology Sarawak, Sibu 96000, Sarawak, Malaysia

**Keywords:** fibric peat, stabilization, microstructural characterization, portland cement, silica fume

## Abstract

Peat is a renowned problematic soil and needs stabilization to enhance its engineering properties. Silica fume (SF) and Ordinary Portland Cement (OPC) were extensively adopted to increase the mechanical properties of peat; however, their microstructural analysis is lacking. Investigated herein is the microstructural evolution caused by the OPC and SF implementation in peat soil stabilization. Initially, the compositional analysis (elements and oxides) of peat and binders was carried out via energy-dispersive X-ray (EDX) and X-ray fluorescence (XRF). Subsequently, the microstructural changes that occurred in the stabilized peat were examined through a series of microstructural analyses. The analysis includes scanning electron microscope (SEM), X-ray diffraction (XRD), Fourier-Transform Infrared Spectroscopy (FTIR), and thermogravimetric analysis (TGA) for morphological, mineralogical, functional group analysis, and bond thermal analysis, respectively. The SEM micrographs evidence the transformation of loosely packed with large micropores of untreated peat into a compact dense peat matrix. This transformation is due to the formation of newly developed minerals, i.e., calcium hydrates (CH), calcium silicate hydrates (C-S-H), calcium aluminate hydrate (CAH), ettringite (Aft) caused by the pozzolanic reaction of binders as recorded by the XRD. Similarly, different molecular functional groups were found in the FTIR analysis with the incorporation of SF and OPC. Finally, the percentage of mass loss was assessed through TGA analysis revealing the decomposition of stabilized in the second and third stages.

## 1. Introduction

Among problematic soils, peat is well-known for its high-water content, weak strength, compressible nature, excessive primary and secondary settlement, and extremely low bearing capacity. However, peatlands cover about 3% of the land surface where the former U.S.S.R and Canada possess the highest about of peatland [1]. In addition to temperate and cold climate zones, peatland is widely spread across the tropical regions of Southeast Asia, i.e., Malaysia and Indonesia. The removal of shallow-depth peat (up to 4 m) or its replacement with competent is often desirable during construction activities on peatland but the excavation, disposal, borrowed materials, etc. becomes expensive and not feasible [2].

Additionally, the removal/replacement of peat involves drainage causing the drawdown of the groundwater table and exposing peat to atmospheric oxygen. Thus, it disturbs the entire hydrological regime of the peatland and initiates aerobic decomposition instead of anaerobic, resulting in high carbon dioxide (CO_2_) discharge into the atmosphere and causing the greenhouse effect [3]. As a result, wetland becomes a massive source of greenhouse gas emissions and jeopardizes their role of being a huge carbon sink [4]. In addition, the drained-out peat having degraded vegetation is highly flammable triggering a catastrophic forest fire emitting a huge amount of CO_2_ into the natural atmosphere [5]. The aforementioned problems impel the boundaries of innovative research to sort out a sustainable solution to utilize wetlands. Thus, a viable peat stabilization instead of removal or replacement is applied.

Several peat stabilization techniques including mechanical, chemical, electrical, biological, etc. stabilizations are currently in practice to enhance their engineering properties to make peatland serviceable [6,7,8,9,10]. Mass stabilization including deep mixing (wet and dry) of potential additives/binders are considered an environmentally friendly and economical solution for peat stabilization [11]. Ordinary Portland Cement (OPC) and lime are the commonly adopted binders; however, utilizing locally available and industrial pozzolanic wastes, i.e., silica fume, fly ash, gypsum, granulated blast furnace slag (GBBS), bentonite, etc. are encouraged [12,13,14,15,16,17,18,19,20,21]. Khanday et al. stabilized peat using RHA-based geopolymer [22] and GBBS [23] and obtained significant strength improvement. Similarly, enormous strength enhancement of peat has been reported by Kalantari et al. [17,24] utilizing cement and silica fume. Since the cement is a hydraulic binder, Kalantari and Prasad [25] assessed the strength development of cement-stabilized peat with prolonged curing age. Similarly, the significant strength improvement and morphological changes of Indian peat caused by cement were reported by Paul and Hussain [18]. Both silica fume and cement-stabilized peat satisfy the minimum strength criteria of 345 kPa. However, for the sake of sustainability, filler materials such as sand, crumbed waste tires, crushed demolished waste, etc. along with cementitious additives are recommended to enhance the weak engineering characteristics of peat by reducing the cement amount [9,25,26]. For this reason, Saberian and Rahgozar [25,26] utilized shredded waste tires and sand as filler materials in cement, lime, and gypsum-stabilized peat. A significant strength enhancement was reported in all combinations, meeting the minimum required strength except for sand-filled peat. Thus, it can be observed in past studies that both cement and silica fume act as potential stabilizers with and without filler materials in peat soil.

Malaysian peat possessing extremely low strength (42.94 kPa) derived from Kampung Baru, Teluk Intan was stabilized by Ahmad et al. [27] using Ordinary Portland Cement (OPC) and silica fume (SF). Apart from indexed properties, mechanical properties including the unconfined compressive strength (UCS) and California bearing ratio (CBR) of the stabilized and untreated peat were assessed and the failure pattern of the tested UCS specimen was examined. A significant amount of strength enhancement was experienced by utilizing OPC and SF. An increasing quantity of binders (OPC and SF) and prolonging the curing duration yielded a higher strength value. The highest strength value of 1063.94 kPa was achieved by SF-stabilized peat after curing for 28 days. Moreover, an acceptable strength has been achieved by OPC and OPC-SF combination in peat. Furthermore, the failure patterns of the UCS samples revealed a ductile behavior which leads to the sustainability application of SF and OPC. However, the reason for the enhanced strength of Malaysian peat stabilized with SF and OPC is not investigated to date. The chemistry behind tremendous strength enhancement due to the use of OPC and SF in Malaysian peat needs to be unveiled.

Due to the lower fraction of clay particles in peat, the strength development of cement and silica fume in peat is questionable. Paul and Hussain [18,28] investigated the mechanical and microstructural performance of cement-stabilized peat. A compact stabilized peat matrix compared to untreated peat was observed in the field emission scanning electron microscopy (FESEM) micrographs. This transformation was further investigated via X-ray diffraction (XRD) by reporting the development of responsible compounds such as calcium silicate hydrate (C-S-H), calcium aluminate hydrate (C-A-H), calcium aluminum silicate hydrate (C-A-S-H), and ettringite. The formation of newly developed compounds due to cement incorporation was also confirmed by thermogravimetric analysis (TGA) and Fourier transform infrared (FTIR) spectroscopy analyses. On the other hand, Rikmann et al. [29] carried out a microstructural analysis on the cement and shale ash based stabilized peat. They came to the conclusion by supporting the utilization of pozzolanic materials such as silica fume, alkali pH modified, and water glass without the addition of OPC in the peat stabilization. It can be observed from the previous studies that the performance of silica fume, cement, and their combination in peat soil is rarely assessed through microstructural tests.

In this context, an intensive microstructural study of OPC and SF stabilized peat is needed for a better understanding of the strength enhancement of the treated Malaysian peat matrix. Therefore, microstructural properties tests including scanning electron microscope (SEM), X-ray diffraction (XRD), Fourier-transform infrared spectroscopy (FTIR), and thermogravimetric analysis (TGA) were carried out in this paper to examine the microstructural evolution in the stabilized peat matrix caused by the hydraulic binders such as cement and silica fume.

## 2. Materials and Experimental Procedure

### 2.1. Peat Details

Peat soil collected from Kampung Baru located in the Teluk Intan state of Malaysia was used in the experimental investigation of this research. The collection site coordinates are 4°00′16.1″ N, 101°11′11.0″ E, and the peat soil was collected at a depth of 5 feet (1.52 m). The in situ von Post humification test categorized Teluk Intan peat as highly fibrous (H_3_) and acidic in nature. The unconfined compressive strength (UCS) of this peat is about 42.94 kPa which is extremely lower than the acceptable strength (345 kPa) [27].

### 2.2. Peat Elemental Analysis (EDX Mapping)

**Figure 1:**
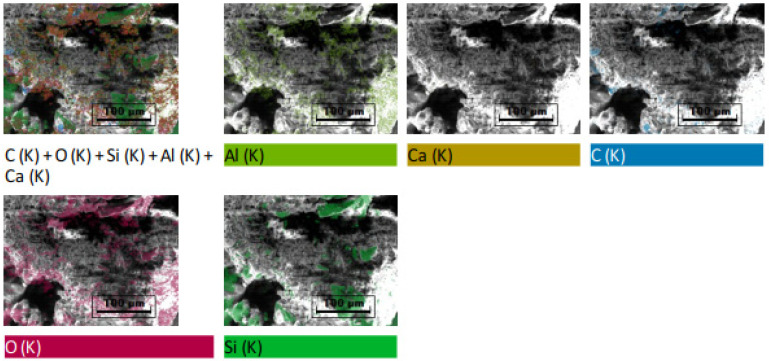
Elemental images of peat from EDX mapping.

An analytical tool was used to chemically characterize the elemental composition of peat known as energy dispersive X-ray (EDX). Table 1 enlists the element present in the peat while Figure 1 illustrates the spectral elemental micrographs of the peat. Being highly organic in nature, peat contains predominantly C (31.88%) and O (46.17%). Moreover, the output revealed that peat also possesses Al (7.83%), Ca (0.80%), and Si (13.32%). The presence of Si and Al is desirable in the baseline soil to induce the pozzolanic reactions [30]. Furthermore, the soil composition influences the selection of binders, therefore, silica fume and OPC being pozzolanic in nature were used as potential peat stabilizers [24].

### 2.3. Properties of Binders

Commonly available Ordinary Portland Cement (OPC) of grade 53 was utilized for the experimentations. OM Materials (Sarawak) Sdn Bhd provides silica fume (SF) which is a byproduct in the silicon and ferrosilicon alloy production during the smelting process (Bintulu, Malaysia). SF is used as a potential binder for peat stabilization in the current research. Its particle is a tiny and spherical shape that is approximately 100 times smaller than cement granules and possesses a diameter of approximately 150 nm. Table 2 enlists the physical and chemical properties of SF provided by the supplier, used in the experimentation. Table 3 illustrates the oxides composition of peat, SF, and OPC.

### 2.4. Experimental Testing Matrix

Table 4 indicates the microstructural experimental matrix including SEM, XRD FTIR, and TGA tests. An oven-dried parent sample and 28 days cured stabilized/treated samples were exposed for testing to reveal the microstructural changes in the peat.

#### 2.4.1. Scanning Electron Microscopy (SEM)

Scanning electron microscopy (SEM) was carried out to investigate and analyze the morphology of the treated and untreated peat derived from Teluk Intan. The Zeiss EVO LS 15 model machine (Oberkochen, Germany) available in the Universiti Teknologi Petronas located in Seri Iskandar, Malaysia was utilized for all the SEM testing. All SEM micrographs were taken from the 28 days cured finely grounded samples in the range of 1000×–10,000× magnification. The mix combinations used for morphological comparison are illustrated in Table 4.

#### 2.4.2. X-ray Diffraction (XRD)

The X-ray diffraction, commonly known as the XRD test was performed to identify the mineralogical composition of untreated and treated Teluk Intan peat. A continuous type PANalytical X’pert powder diffractometer (Malvern, UK), available in the Central Analytical Laboratory (CAL) of the Universiti Teknologi Petronas (UTP) was utilized keeping the scan range of 10–90° and a step of 0.0262606°. A finely grounded XRD sample smaller than 75 μm was exposed to a radiation source of CuKα having the wavelength (λ) of 1.5418 A° with an input voltage of 40 kV and a current of 30 mA. All the XRD analysis has been performed on all four mixes: parent peat and three stabilized peat samples, i.e., peat + SF, peat + OPC, and peat + OPC + SF as described in Table 4.

#### 2.4.3. Fourier-Transform Infrared Spectroscopy (FTIR)

The Fourier-transform infrared spectroscopy (FTIR) test was performed to investigate the chemical bonds or the functional groups in the untreated and treated peat derived from Teluk Intan. For this purpose, a PerkinElmer model spectrometer, installed at the Universiti Teknologi Petronas (UTP) located in Seri Iskandar, Perak state of Malaysia was utilized (Waltham, MA, USA). It is equipped with a diamond attenuated total reflectance (ATR) having a scanning range of about 500–4000 cm^−1^ with a 4 cm^−1^ resolution. For the test to perform, a mixture of about 5 mg of finely grounded peat and 200 mg of KBr were exposed to an infrared spectrum. The FTIR analysis was performed on the untreated peat, peat + SF mix, peat + OPC mix, and peat + OPC + SF mix after 28 days of curing as illustrated in Table 4.

#### 2.4.4. Thermogravimetric Analysis (TGA)

PerkinElmer STA 6000 simultaneous thermal analyzer available at the Central Analytical Laboratory (CAL) of the Universiti Teknologi Petronas (UTP) was employed to perform the thermogravimetric analysis (TGA) of treated and untreated peat as described in Table 4. All the TGA tests were performed at a temperature ranging from 30 °C to 800 °C with an increment of 10 °C/min under a nitrogenous environment.

## 3. Results and Discussion

### 3.1. Scanning Electron Microscopy (SEM)

**Figure 2:**
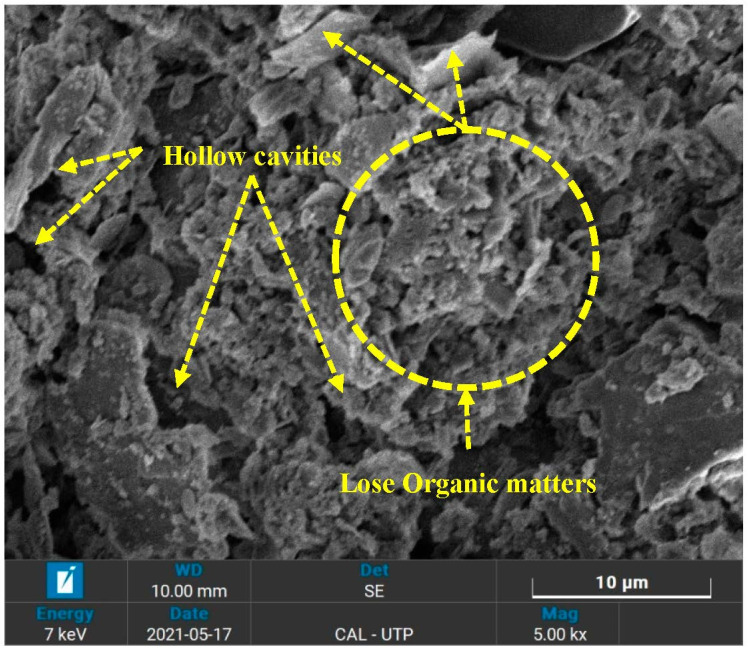
SEM image of untreated peat.

Scanning electron microscopy (SEM) was carried out to examine the morphology of a treated and untreated peat derived from Teluk Intan, Perak. The internal mineralogical formation of peat significantly alters upon the application of OPC, and SF. Figure 2 illustrates a 300× magnified SEM image of untreated peat. It can be seen that the internal structure of the untreated peat is made up of hollow cavities and pores, flaky and loosely packed, and spongy organic matter. Typically, the organic matters are hollow from the inside and spongy in nature and hence possesses high water-holding capacity upon saturation [31,32]. Moreover, the entire topography is arranged randomly, without profound orientation. The same morphology of Malaysian peat has been reported by other researchers [32,33,34,35,36,37]. These factors are responsible for the low unconfined compressive strength (UCS) and California bearing ratio (CBR) values of the untreated peat as reported by Ahmad et al. [27].

**Figure 3:**
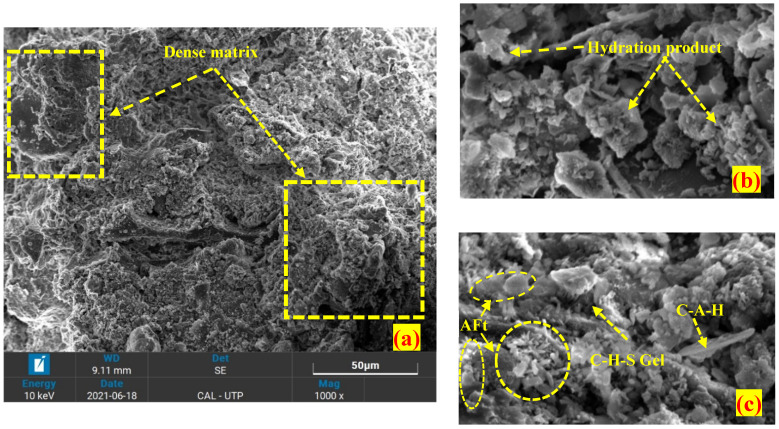
SEM image of 28-day cured OPC stabilized peat (**a**) 1000×, (**b**) 8000×, (**c**) 10,000×.

**Figure 4:**
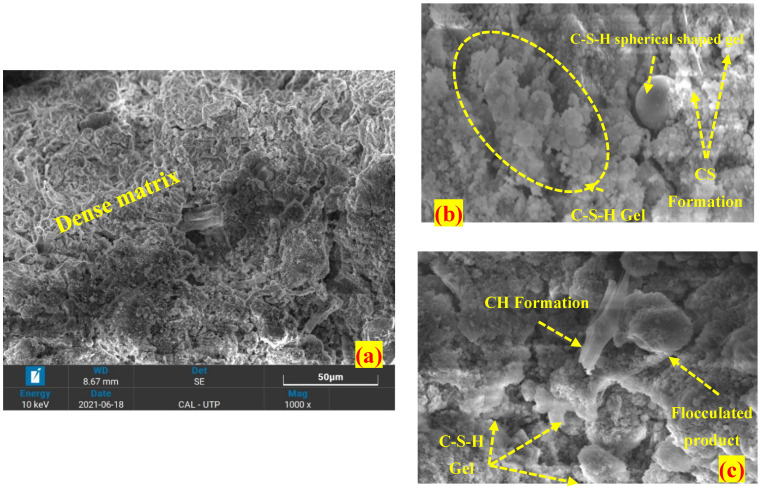
SEM images of 28-day cured SF stabilized peat (**a**) 1000×, (**b**) 8000×, (**c**) 10,000×.

**Figure 5:**
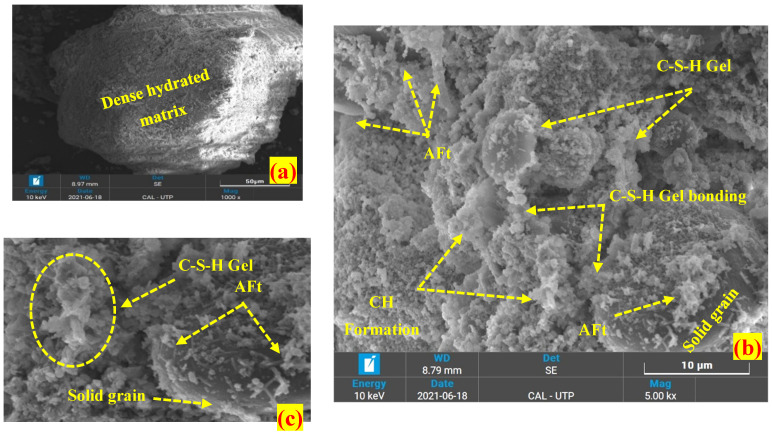
SEM images of 28 days cured OPC-SF stabilized peat (**a**) 1000×, (**b**) 5000×, (**c**) 10,000×.

On the other hand, the 28-day cured OPC stabilized peat has a dense and compacted morphology compared to untreated peat, as seen in Figure 3. The development of C-S-H gel, C-A-H, AFt, and micropores reduction as shown in Figure 3b,c reasonably enhances the strength of peat [33,38,39]. Similar products have been observed in the silica fume-stabilized peat. However, the silica fume (SF) stabilized peat seems more uniformly compacted compared to the OPC stabilized peat as illustrated in Figure 4a,b. The utilization of both OPC and SF in a single mix creates a denser matrix after 28 days of curing by filling the pores more efficiently as observed in Figure 5a. Moreover, cementitious products, i.e., C-S-H gel, AFt, and C-A-H formation have been observed noticeably as seen in Figure 5b,c. Thus, they gained higher UCS and CBR values compared to OPC-treated and silica fume-treated peat samples [27].

Overall, the application of OPC, SF, and OPC-SF reduces the pores and improves the surficial characteristics by filling the void spaces and binding the peat components into a dense flocculated mass. Moreover, the development of calcium silicate hydrate (C-S-H), calcium aluminate hydrate (C-A-H), aluminum calcium oxide (C-A-O), and ettringites (AFt) are the indication of a strong interfacial bonding network which helps in the interlocking and friction coefficient among the soil particles [18,31]. These are the reasons treated Teluk Intan peat exhibits higher UCS and CBR values compared to untreated peat samples [27]. Moreover, some packs of grain particles have been observed which may be formed due to the presence of water and clay coagulations.

### 3.2. X-ray Diffraction (XRD)

**Figure 6:**
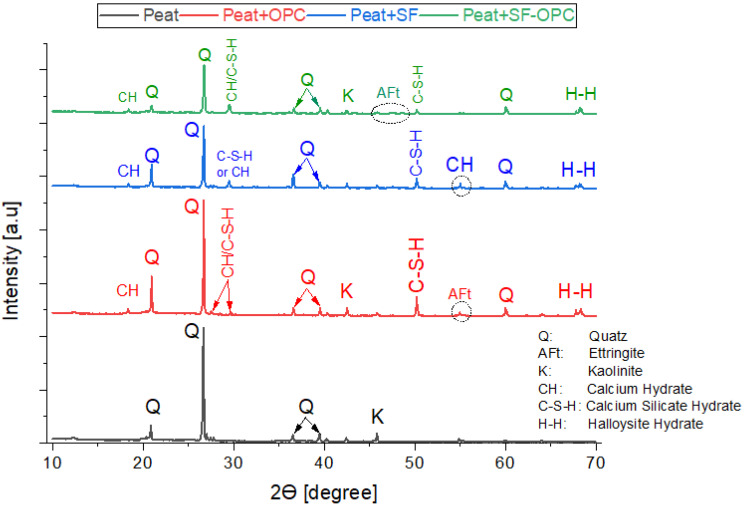
XRD analysis of Peat only, Peat + OPC, Peat + SF, and Peat + OPC + SF.

The X-ray diffraction (XRD) was carried out to identify the minerals as well as reveal the pozzolanic activities in stabilized peat upon being treated with different additives. Figure 6 illustrates the XRD results of parent peat, peat treated with OPC, silica fume, and the combination of OPC and silica fume, respectively. The interpreted XRD results of the stabilized peat were performed on the 28-day cured specimen.

The XRD results of parent peat (untreated) are shown in Figure 6. It is observed that quartz (SiO_2_) is the primary mineral present in the untreated peat along with clay minerals such as kaolinite (Al_2_O_3_ 2SiO_2_·2H_2_O). The prominent peaks of quartz were found at the position of 2θ (20.93°, 26.71°, 36.64°, 39.55°, 59.98°) and kaolinite at 2θ position of 40.29° and 42.52°. The existence of quartz and kaolinite in the same position was evident in the previous studies [30,40,41]. Furthermore, the existence of quartz in peat has been evidenced by Khanday et al. [42,43] and clay minerals by Paul and Hussain [28]. Moreover, Moayedi et al. [44] particularly mentioned kaolinite as the prominent clay mineral present in untreated peat soil.

Similarly, the application of Ordinary Portland Cement (OPC) and silica fume impart some new minerals which are considered responsible for the stabilization. As seen in Figure 6, ettringite (AFt phase), calcium hydrate [Ca (OH)_2_], calcium silicate hydrate (5Ca_2_SiO_4_·6H_2_O), calcium aluminate hydrate (3Ca_2_Al_2_O_3_·6H_2_O), and halloysite hydrate [Al2Si_2_O_5_(OH)_4_·2H_2_O] are the prominent detected minerals upon using OPC and silica fume as a stabilizing agent. Compared with the untreated peat, the reduction in peak intensities as illustrated in Figure 6 is attributed to the aforementioned mineral’s formation due to the pozzolanic reactions of OPC and silica fume, respectively [45].

### 3.3. Fourier-Transform Infrared Spectroscopy (FTIR)

**Figure 7:**
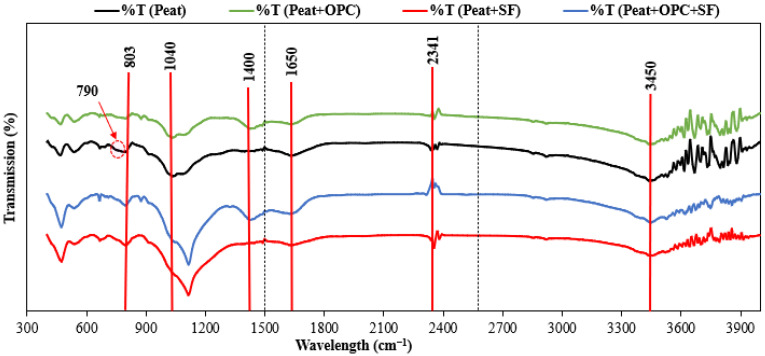
FTIR analysis of the untreated peat and treated peat with OPC, SF, and OPC + SF.

The evidence of the different molecular functional groups in the parent and treated Teluk Intan peat is illustrated in Figure 7 and Table 5. Like Paul and Hussain [18], the entire absorption band series has been divided into three regions to analyze the data, i.e., 4000–2500 cm^−1^, 2500–1500 cm^−1^, and 1500–400 cm^−1^ [46].

As shown in the first region of Figure 7, a single known absorption peak has been noticed at 3450 cm^−1^ which corresponds to the stretching of the O-H group. In the second region, two prominent peaks were observed at 2341 and 1650 cm^−1^. Along 2341 cm^−1^, the intensities increase in the SF and OPC + SF treated peat which indicates the development of –C=O stretching of the aliphatic compound. The double bond of cyclic compounds was evident at 1650 cm^−1^, especially in OPC-treated peat [28,46]. Similarly, the occurrence of peaks in the last absorption band was found at 1400, 1040, 803, and 790 cm^−1^. A prominent absorption peak has been identified at 1400 cm^−1^ in the cement-treated peat spectra (i.e., Peat + OPC and Peat + OPC + SF) due to the development of Ca-OH bond upon the reaction of atmospheric CO_2_ and CH during the curing of samples as reported by [28,47,48]. The occurrence of polysaccharide, C=C bond, and Si-O stretching are represented by 1040, 803, and 790 cm^−1^ peaks [28,49,50]. Moreover, the intense peaks were observed around 1118 cm^−1^ in the SF-treated peat samples, i.e., Peat + SF and Peat + OPC + SF. For this, Yacob and Som [48] mentioned the development of C-S-H in the absorption band at 1100–1200 cm^−1^. Hence, the FTIR results confirmed the development of new compounds which imparts considerable changes in the absorption bands when treated peat with OPC and SF.

### 3.4. Thermogravimetric Analysis (TGA)

**Figure 8:**
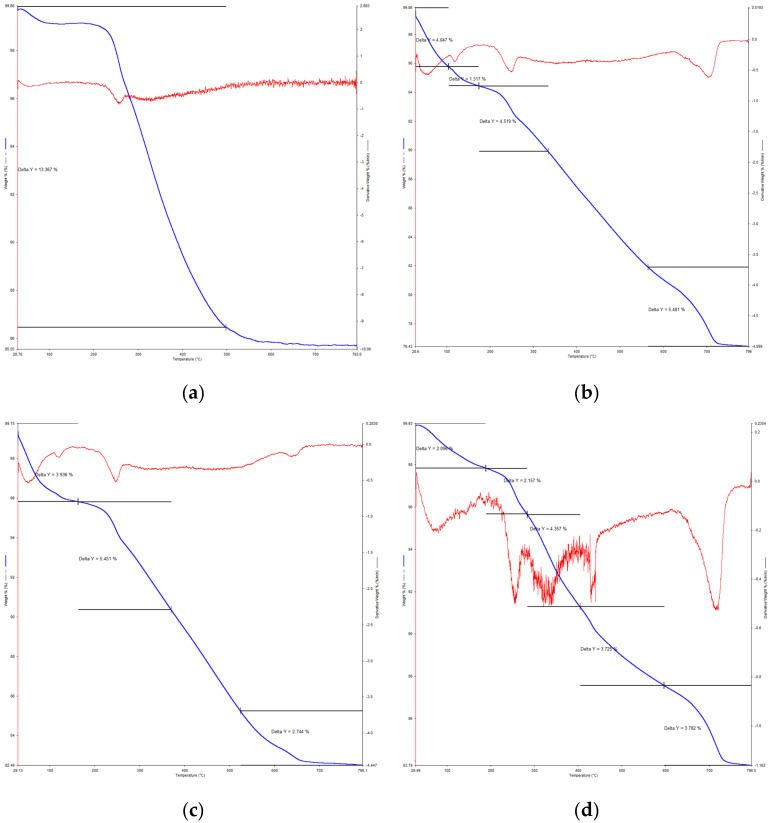
Thermogravimetric analysis of treated and untreated peat. (**a**) Peat, (**b**) Peat + OPC, (**c**) Peat + SF, and (**d**) Peat + OPC + SF.

The thermal degradation of untreated and treated peat was carried out using the TGA test. Similar to the other microstructural tests, untreated and three treated and cured samples were employed for the TGA testing including Peat, Peat + OPC, Peat + SF, and Peat + OPC + SF. The presented results shown in Figure 8 have two curves: the blue line indicates the mass loss vs. temperature (TGA curve), and the red line indicates the first derivative of mass loss vs. temperature (DTG curve).

Past studies reported that peat undergoes pyrolytic decomposition due to its complex qualitative and quantitative compositions [51,52]. Moreover, it has been observed in the previously published articles that peat decomposition occurred at three different stages. Initially, the dehydration of bound (hygroscopic) and free (capillary) water occurs at a temperature range of 27–150 °C. The capillary water evaporates during oven drying while the water present in the hydrated products, i.e., C-S-H gel and C-A-H of stabilized peat evaporates during the TG test at elevated temperature [51,53,54]. On the other hand, the mass loss in the second stage (150–600 °C) is attributed to the less condensed components such as the components of humic acids as well as aliphatic compounds functional groups [55]. Comparing the parent peat DTG curve shown in Figure 8a with the rest of the stabilized peat curves illustrated in Figure 8b–d after 300 °C, prominent mass loss as is noticed in the form of endothermic and exothermic peaks. However, beyond 600 °C temperature, there are more condensed materials, i.e., ettringite (Aft), aromatic components of lignin as well as the humic acids nuclear region [56].

## 4. Conclusions and Recommendations

This study investigated the microstructural characteristics of untreated and stabilized Malaysian fibric peat caused by the incorporation of SF and OPC. Several microstructural tests including the scanning electron microscope (SEM), X-ray diffraction (XRD), Fourier-Transform Infrared Spectroscopy (FTIR), and thermogravimetric analysis (TGA) were carried out to assess the microstructural changes in the stabilized peat caused by SF and OPC. The following conclusions have been drawn.

1. The morphological test (SEM) exposed hollow cavities/pores, spongy organic matters, and the flaky and loosely packed internal structure of the parent peat which is responsible for its low compressive and bearing capacity. On the other hand, a compact matrix with strong interparticle bonding is revealed in the SF and OPC stabilized peat.
2. The morphological alteration in the stabilized peat is further investigated in XRD and observed in the formation of a newly developed compound. The hydration products, i.e., calcium hydrate (CH) and calcium silicate hydrate (C-S-H) are dominantly found in the SF-treated peat. Similarly, the Aft formation, calcium hydrate (CH), calcium silicate hydrate (C-S-H), and calcium aluminate hydrates (CAH) are the prominent products found in the OPC and SF-OPC treated peat.
3. The prominent functional groups were observed during FTIR spectral analysis in the stabilized peat including Si-O, –C=O, O-H stretching, and C=C and Ca-OH bond formation.
4. The TG analysis revealed that untreated peat decomposes in the first stage (27–150 °C). Most of the hydrated products in the stabilized peat undergo decomposition in the second stage of heating (150–600 °C). Moreover, the ettringite (Aft) developed as a result of OPC incorporation decomposes beyond 600 °C.
5. This study revealed the microstructural changes that occurred in Teluk Intan peat using an industrial stabilizer SF and OPC. While the mechanical aspect of the same peat using SF and OPC is evaluated by Ahmad et al. [27]. However, the environmental effect of industrial waste (SF) and OPC in peat is still lacking and needs to be assessed.

## Figures and Tables

**Table 1:** Quantitively elemental analysis of peat.

Element	Atomic (%)	Weight (%)
Aluminum	4.59	7.83
Calcium	0.31	0.80
Carbon	41.97	31.88
Oxygen	45.62	46.17
Silicon	7.50	13.32

**Table 2:** Physical and chemical characteristics of SF.

Properties	Value/Description
Appearance	Ultrafine amorphous powder
Colour	Grey, off-white
Odor	Odorless
pH @ 20 °C	6.0–9.0
Solubility (water)	Insoluble/slightly soluble
Solubility (Organic solvents)	Insoluble/slightly soluble
Melting point	1550–1700 °C
Bulk density	150–700 kg/m^3^
Specific gravity	2100–2300 kg/m^3^
Particle Size	0.4–0.5 μm

**Table 3:** XRF analysis of peat, SF, and OPC.

Oxides (% Weight)	Peat	SF	OPC
CO_2_	90.12	-	-
Al_2_O_3_	0.746	0.876	2.68
SiO_2_	6.64	91.0	12.3
CaO	0.355	1.20	75.5
Fe_x_O_y_	0.673	3.39	2.86
K_2_O	0.045	1.42	0.479
TiO_2_	0.020	0.013	0.231
SO_3_	0.942	0.352	1.32
MgO	0.142	0.409	1.80
P_2_O_5_	0.030	-	-
ZrO_2_	-	0.075	0.217
MoO_3_	0.24	0.052	1.29
MnO	-	0.205	0.411
ZnO	0.032	0.0292	0.0275
Total weight (%)	99.98	99.02	99.10

**Table 4:** Microstructural testing matrix.

Combination	SEM	XRD	FTIR	TGA
Peat	✓	✓	✓	✓
Peat + OPC	✓	✓	✓	✓
Peat + SF	✓	✓	✓	✓
Peat + OPC + SF	✓	✓	✓	✓

**Table 5:** Tentative band assignment for IR spectra of parent and stabilized peat.

Wavelength (cm^−1^)	Tentatively Assigned Band
3450	O-H stretching
2341	–C=O stretching of the aliphatic compound
1650	C=C of cyclic compounds
1400	Ca-OH bond formation
1040	Polysaccharide occurrence
803	C=C bond formation
790	Si-O stretching

## Data Availability

Not applicable.
